# The influence of feeding linoleic, gamma-linolenic and docosahexaenoic acid rich oils on rat brain tumor fatty acids composition and fatty acid binding protein 7 mRNA expression

**DOI:** 10.1186/1476-511X-7-45

**Published:** 2008-11-16

**Authors:** Javad Nasrollahzadeh, Fereydoun Siassi, Mahmood Doosti, Mohammad Reza Eshraghian, Fazel Shokri, Mohammad Hossein Modarressi, Javad Mohammadi-Asl, Khosro Abdi, Arash Nikmanesh, Seyed Morteza Karimian

**Affiliations:** 1 Department of Nutrition and Biochemistry, Tehran University of Medical Sciences, Tehran, Iran; 2 Department of Clinical Biochemistry, Tehran University of Medical Sciences, Tehran, Iran; 3 Department of Biostatistics, Tehran University of Medical Sciences, Tehran, Iran; 4 Department of Immunology, Tehran University of Medical Sciences, Tehran, Iran; 5 Department of Medical Genetics, Tehran University of Medical Sciences, Tehran, Iran; 6 Department of Medicinal Chemistry and Pharmaceutical Sceinces, Tehran University of Medical Sciences, Tehran, Iran; 7 Department of Pathology of Shariati Hospital, Tehran University of Medical Sciences, Tehran, Iran; 8 Department of Physiology, Tehran University of Medical Sciences, Tehran, Iran

## Abstract

**Background:**

Experimental studies indicate that gamma linolenic acid (GLA) and docosahexaenoic acid (DHA) may inhibit glioma cells growth but effects of oral consumption of these fatty acids on brain tumor fatty acid composition have not been determined in vivo.

**Methods:**

GLA oil (GLAO; 72% GLA), DHA oil (DHAO; 73% DHA) were fed to adult wistar rats (1 mL/rat/day) starting one week prior to C6 glioma cells implantation and continued for two weeks after implantation. Control group were fed same amount of high linoleic acid safflower oil (74–77% linoleic acid). Fatty acid composition of tumor samples was determined in a set of 8–12 animals in each group and serum fatty acid in 6 animals per each group. Gene expression of tumor fatty acid binding protein 7 (FABP7), epidermal growth factor receptor (EGFR), peroxisome proliferator activated receptor γ (PPAR-γ) and retinoid × receptor-α (RXR-α) were determined in a set of 18 animals per group.

**Results:**

DHAO feeding increased EPA of brain tumors and decreased ratio of n-6/n-3 fatty acids. Serum levels of EPA were also increased in DHAO group. A similar trend in serum and tumor levels of DHA were observed in DHAO group but it did not achieve statistical significance. GLAO increased serum concentration of GLA but had no significant effect on tumor GLA or dihomo-gamma linolenic acid (DGLA) concentrations. Gene expression of FABP7 was up-regulated in tumors of DHAO group but no other significant effects were observed on EGFR, PPAR-γ or RXR-α expression, and expression of these genes in tumors of GLAO were not different from SFO group.

**Conclusion:**

Dietary supplementation of DHA containing oil could be an effective way to increase levels of long chain n-3 fatty acids in brain tumors and this increase may be mediated partly by up-regulation of FABP7 expression.

## Background

Polyunsaturated fatty acids (PUFAs) have diverse functions in living cells and influence membrane composition and function, eicosanoid synthesis, cellular signaling and regulation of gene expression [1]. Experimental studies have shown that supplementation of tumor cells with specific fatty acids may decrease tumor proliferation. Gamma-linolenic acid (GLA), an n-6 fatty acid, and n-3 fatty acids such as eicosapentaenoic acid (EPA) and docosahexaenoic acid (DHA) have received considerable attention due to their anti-tumor activity against many tumor cell lines [2-4]. Previous in vivo and clinical studies have reported that GLA infusion into the rat or human glioma could be effective in decreasing tumor growth [2,3,5,6]. Although effective, this method can not be applied for majority of patients. Long chain PUFAs of n-3 fatty acid such as EPA and DHA has also been shown to have anti-proliferative effect on glioma cells in vitro [7,8]. Dietary intake of GLA and n-3 fatty acids has been explored in some animal model of prostate, breast and colon cancers [4,9-12] but these fatty acids may not be as effective in brain tumors since blood brain barrier can limit their uptake by brain tumor cells. Since dietary intake of PUFAs could influence normal brain structure and biological function [13], it may also affect brain tumor fatty acid composition and function. A few studies with limited samples have compared fatty acid composition of human brain tumors with that of normal brain tissue and have found that concentration of n-3 fatty acids, especially DHA, are lower in gliomas than non-tumoral brain tissues [14,15].

Uptake and transport of PUFAs across cellular membrane occurs to some extend by passive diffusion and additionally is facilitated by a number of membrane associated and cytoplasmic proteins[16]. Fatty acid binding proteins (FABPs) are thought to facilitate transport and intracellular trafficking of fatty acids [16]. FABP7 (brain fatty acid binding protein) is a member of FABPs which is highly expressed in glial cells and bind to long-chain PUFA with high affinity [17,18]. Its expression in glioma cell lines and brain tumor specimens has been reported [19,20]. Additionally, in human brain tumor specimens, FABP7 expression has been shown to be associated with epidermal growth factor receptor (EGFR) overexpression [21].

PPAR-γ and RXR-α are members of nuclear receptors that have been shown to be activated by some PUFAs [22,23]. In vitro and in vivo studies demonstrated that ligands of these receptors may be effective in reducing growth and invasiveness of glioma cell lines [24,25].

Rat C6 glioma model has been proven to be useful for a variety of studies related to brain tumor biology [26]. The aim of this study was to investigate the effects of oral feeding of oils containing high concentration of GLA, DHA or linoleic acid on blood and tumor total fatty acid composition in rat C6 glioma model. In addition, we studied effects of supplementation of these oils on gene expression of FABP-7, EGFR, RXR-α and PPAR-γ as molecular targets of PUFAs through which PUFAs may influence functions of glioma tumors.

## Results

### Serum and tumor sample fatty acid composition

Table 1 shows serum fatty acid levels of rats. Concentration of GLA significantly increased in GLAO group relative to SFO (5.1 fold; *p *< 0.05) or DHAO (13.3 fold; *p *< 0.01) groups. In DHAO group, EPA levels were much greater than SFO (21.8; *p *< 0.01) or GLAO (27.1 fold; *p *< 0.01) groups. In addition, sum of n-3 fatty acids were significantly higher and ratios of arachidonic acid/DGLA+EPA and n-6/n-3 were significantly lower in DHAO group compared to the two other groups. Levels of DHA were significantly higher in DHAO than GLAO (5.8 fold; *p *< 0.05) but the increase in DHAO did not achieve statistical significance when compared to SFO group (2.8 fold, *p *value = 0.10). Arachidonic acid levels and sum of n-6 fatty acids were also lower in DHAO relative to SFO group (*p *< 0.01 and *p *< 0.05 respectively).

**Table 1:** Fatty acid levels of rat serum of SFO, GLAO and DHAO groups

**Fatty acids**	**Concentration in serum (μg/mL)**			**one way ANOVA**
		
	**SFO**	**GLAO**	**DHAO**	
Miristic Acid (C14:0)	19.46 ± 0.44	19.76 ± 0.56	21.17 ± 1.24	NS

Palmitic Acid (C16:0)	89.28 ± 19.99	51.26 ± 12.72	49.83 ± 9.39	NS

Stearic Acid (C18:0)	99.86 ± 22.20	72.89 ± 21.60	62.69 ± 14.81	NS

Arachidic Acid (C20:0)	2.06 ± 0.00	2.22 ± 0.15	2.76 ± 0.69	NS

Behenic Acid (C22:0)	11.27 ± 0.60	10.73 ± 0.15	11.38 ± 0.39	NS

Oleic Acid(C18:1, n-9)	55.56 ± 14.91	23.44 ± 8.19	30.49 ± 7.53	NS

11-Ecosenoic Acid(C20:1)	52.04 ± 30.86	84.67 ± 57.88	30.19 ± 25.44	NS

Nervonic Acid (C24:1)	5.72 ± 00.00	5.99 ± 0.27	5.72 ± 00.00	NS

Linoleic Acid(C18:2, n-6)	229.75 ± 79.27	61.40 ± 18.03	94.16 ± 32.64	NS

Gamma linolenic acid(C18:3, n-6)	11.46 ± 6.55^a^	59.34 ± 16.44^b^	4.46 ± 2.75^a^	*P *< 0.01

11,14-Ecosadienoic Acid (C20:2, n-6)	trace	0.54 ± 0.76	1.90 ± 1.27	NS

Dihomo-Gamma Linolenic Acid(C20:3, n-6)	2.65 ± 0.50	4.137 ± 0.84	2.71 ± 0.52	NS

Arachidonic Acid(C20:4, n-6)	234.42 ± 45.92^a^	151.13 ± 34.27^ab^	36.54 ± 8.68^b^	*P *< 0.01

13,16-Docosadienoic Acid (DDA, C22:2, n-6)	6.21 ± 0.39	5.81 ± 0.22	6.00 ± 0.29	NS

Alpha-Linolenic Acid(C18:3, n-3)	trace	trace	0.26 ± 0.20	NS

11,14,17-Eicosatrienoic Acid (C20:3, n-3)	2.61 ± 0.04	2.61 ± 0.06	2.56 ± 0.01	NS

Eicosapentaenoic acid (C20:5, n-3)	3.28 ± 1.64^a^	2.60 ± 1.31^a^	70.51 ± 17.66^b^	*P *< 0.001

Docosahexaenoic acid (C22:6, n-3)	38.27 ± 5.92^ab^	18.48 ± 4.22^a^	107.31 ± 37.62^b^	*P *< 0.05

Arachidonic/Dihomo-Gamma Linolenic + Eicosapentaenoic	83.77 ± 48.46^a^	26.75 ± 3.25^a^	0.56 ± 0.09^b^	*P *< 0.001

Sum of n-6 fatty acids *	484.47 ± 118.15^a^	282.36 ± 65.79^ab^	145.78 ± 41.41^b^	*P *< 0.05

Sum of n-3 fatty acids^§^	44.26 ± 6.10^a^	23.70 ± 5.42^a^	180.66 ± 53.10^b^	*P *< 0.001

n-6 to n-3 Ratio^‡^	10.38 ± 1.62^a^	11.84 ± 1.11^a^	0.85514 ± 0.07^b^	*P *< 0.001

* Including C18:2, C18:3, C20:2, C20:3, C20:4, C22:2. ^§ ^Including C18:3, C20:3, C20:5, C22:6. ^‡ ^Ratio of sum of n-6 fatty acids to sum of n-3 fatty acids. Values are shown as mean ± S.E., n = 6 for each group. ANOVA: Analysis of variance. NS: not significant. Groups not sharing the same letters are significantly different (*Post-hoc *Tukey's test)

Table 2 shows fatty acid profile of rats' tumor tissues. Fatty acid analysis was performed on 8 samples of SFO group because one sample was lost during lipid extraction. The EPA content in DHAO group was greater than SFO (6 fold; *p *< 0.01) and GLAO (5.3 fold (*p *< 0.01), and sum of n-3 fatty acid levels were significantly higher in DHAO than SFO (2.2 fold; *p *< 0.05) and GLAO (3 fold; *p *< 0.01) groups. Ratios of Arachidonic acid/DGLA+EPA and n-6/n-3 were lower in DHAO group compared to the two other groups. Levels of GLA were not significantly different between three groups but linoleic acid content decreased in GLAO relative to SFO group (2.8 fold; *p *< 0.01). Similar to serum, DHA levels were significantly higher in DHAO relative to GLAO (2.8 fold; *p *< 0.05) but non-significantly higher in DHAO than in SFO group (1.9 fold; *p*= 0.13).

**Table 2:** Fatty acid levels of rat tumors of SFO, GLAO and DHAO groups

**Fatty acids**	**Concentration in tumors (μg/g)**			**one way ANOVA**
		
	**SFO**	**GLAO**	**DHAO**	
Miristic Acid(C14:0)	30.57 ± 4.22	35.51 ± 6.00	46.93 ± 9.13	NS

Palmitic Acid (C16:0)	1142.71 ± 284.20	1421.68 ± 302.96	1841.48 ± 408.66	NS

Stearic Acid (C18:0)	2131.56 ± 648.05	1593.07 ± 303.45	2236.11 ± 465.52	NS

Arachidic Acid (C20:0)	12.80 ± 5.74	16.38 ± 4.19	27.43 ± 7.49	NS

Behenic Acid (C22:0)	27.79 ± 5.71^a^	104.28 ± 37.76^ab^	211.62 ± 66.79^b^	*P *< 0.01

Oleic Acid(C18:1, n-9)	1368.56 ± 284.52	1787.10 ± 409.95	2793.21 ± 688.84	NS

11-Ecosenoic Acid(C20:1)	237.95 ± 103.44	262.70 ± 61.21	228.70 ± 65.03	NS

Nervonic Acid (C24:1)	7.45 ± 1.73	31.07 ± 14.25	20.12 ± 10.90	NS

Linoleic Acid(C18:2, n-6)	423.02 ± 79.54^a^	148.69 ± 24.49^b^	259.145 ± 48.66^ab^	*P *< 0.01

Gamma linolenic acid(C18:3, n-6)	13.10 ± 11.02	39.57 ± 11.93	32.15 ± 13.39	NS

11,14-Ecosadienoic Acid (C20:2, n-6)	80.29 ± 60.90	31.89 ± 11.76	32.38 ± 8.46	NS

Dihomo-Gamma Linolenic Acid(C20:3, n-6)	32.29 ± 8.95	55.91 ± 16.27	40.98 ± 13.11	NS

Arachidonic Acid(C20:4, n-6)	1034.95 ± 285.23	970.49 ± 163.15	724.44 ± 142.53	NS

13,16-Docosadienoic Acid (DDA, C22:2, n-6)	18.87 ± 9.49	25.23 ± 12.66	28.79 ± 8.49	NS

Alpha-Linolenic Acid(C18:3, n-3)	trace	trace	0.80 ± 0.40	NS

11,14,17-Eicosatrienoic Acid (C20:3, n-3)	7.50 ± 2.61	6.93 ± 1.88	10.74 ± 2.33	NS

Eicosapentaenoic acid (C20:5, n-3)	58.67 ± 16.49^a^	67.30 ± 15.79^a^	359.12 ± 87.63^b^	*P *< 0.01

Docosahexaenoic acid (C22:6, n-3)	746.72 ± 289.75^ab^	507.61 ± 110.12^a^	1423.24 ± 329.32^b^	*P *< 0.05

Arachidonic/Dihomo-Gamma Linolenic + Eicosapentaenoic	9.34 ± 2.22^a^	10.84 ± 2.07^a^	2.38 ± 0.46^b^	*P *< 0.01

Sum of n-6 fatty acids *	1608.55 ± 302.03	1271.81 ± 208.86	1117.89 ± 187.10	NS

Sum of n-3 fatty acids^§^	813.16 ± 301.04^a^	581.86 ± 121.50^a^	1793.92 ± 383.56^b^	*P *< 0.01

n-6 to n-3 Ratio^‡^	4.12 ± 1.41^a^	3.07 ± 0.73^a^	0.72 ± 0.09^b^	*P *< 0.05

* Including C18:2, C18:3, C20:2, C20:3, C20:4, C22:2. ^§ ^Including C18:3, C20:3, C20:5, C22:6. ^‡ ^Ratio of sum of n-6 fatty acids to sum of n-3 fatty acids. Values are shown as mean ± S.E., n = 8 for SFO group, n = 12 for GLAO group, and n = 9 for DHAO group. ANOVA: Analysis of variance. NS: not significant. Groups not sharing the same letters are significantly different (*Post-hoc *Tukey's test)

### Gene expression

**Figure 1:**
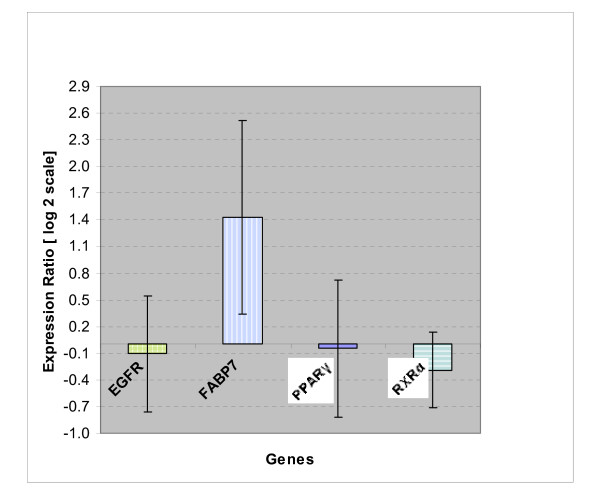
**Expression ratio of genes in DHAO group**. Supplementation of DHAO increased gene expression of brain tumors FABP7 of rats relative to SFO group (*p *< 0.05). Log_2 _scale of expression ratios and singnificancy were calculated by *REST*, using beta-actin and GAPDH as reference genes and tumor samples of SFO group as control group and amplification efficiencies were considered in the analysis. Each value is mean ± S.E. of eighteen determination performed on separate animals.

Figure 1 shows relative expression ratio of FABP7, EGFR, PPAR-γ and RXR-α in brain tumors of DHAO when compared to SFO group. Only FABP7 expression was significantly changed and up-regulated in DHAO group relative to SFO (P < 0.05) and the other variation in gene expression were not significantly different.

**Figure 2:**
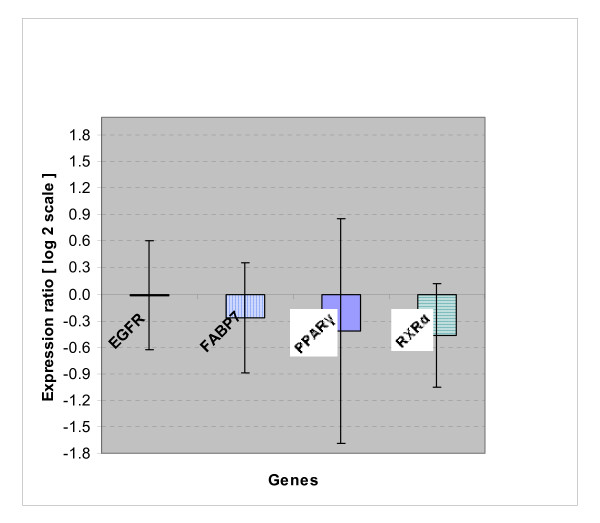
**Expression ratio of genes in GLAO group**. Supplementation of GLAO had no significant effects on gene expression of EGFR, FABP7, PPAR-γ and RXR-α relative to SFO group. Log_2 _scale of expression ratios and singnificancy were calculated by *REST*, using beta-actin and GAPDH as reference genes and tumor samples of SFO group as control group and amplification efficiencies were considered in the analysis. Each value is mean ± S.E. of eighteen determination performed on separate animals.

Figure 2 shows relative expression ratio of genes in GLAO group relative to SFO group. No significant differences in expression of FABP7, EGFR, PPAR-γ or RXR-α were detected in GLAO when compared to SFO group.

## Discussion

Results of current study show that feeding of DHA containing oil to rats could increase EPA content of their brain tumors. We expected it to also increase DHA levels of tumor tissues but DHA levels only increased relative to GLAO group. However, the data did show a trend to higher levels of DHA in tumors of DHAO group compared to SFO group. Similar results observed in serum levels of DHA in studied groups. Previous works have found that fatty acids in ethyl ester form have lower digestive and absorptive efficiency than triglycerides [27] but we do not believe slower digestion or absorption of ethylated DHA was the main cause of non-significant increase of serum or tumor DHA because serum EPA concentration increased in DHAO group. Since EPA content of feeding oil was very low, apparent increase in EPA content of serum and tumor might be due to retroconversion of DHA to EPA. Retroconversion, a peroxisomal process involving one cycle of β-oxidation, has been reported in the liver of rats given a single dose of DHA [28] and in humans consuming DHA [28,29]. In this study, serum concentration of EPA increased in rats given DHAO which implicate retroconversion might have occurred in peripheral tissues such as liver [30,31].

Serum levels of arachidonic acid decreased in DHAO relative to SFO group but decrease in tumor arachidonic acid of DHAO group did not reach to level of significance. DGLA, arachidonic acid and EPA are precursors for series 1, 2 and 3 of prostanoids respectively. Increase in EPA levels and decrease in arachidonic acid/DGLA+EPA levels in DHAO group could influence balance of prostaglandins and leukotriens produced in tissue toward less inflammatory eicosanoids [9,10,32]. In addition, decrease in ratio of n-6/n-3 fatty acids in DHAO group indicate that consumption of DHA may results in reciprocal replacement of n-6 by n-3 fatty acids in tumor. In DHAO group, levels of behenic acid (C22:0) increased, the origin or importance of this increase is not known.

Serum levels of GLA in GLAO group increased but no significant increase in GLA levels of tumor tissues of GLAO group were observed. The lack of increase in the GLA level of tumor indicate that it may not have high uptake by brain tumors or it may has been converted to longer chained fatty acids in the tissue. In an study on male rats, consumption of diet containing borage oil (comprised of 21% GLA and 35.8% linoleic acid) for two weeks increased serum GLA, DGLA and arachidonic acid levels and brain DGLA and arachidonic acid levels relative to corn oil diet (brain GLA concentrations were not determined) [33]. In our study, neither serum nor brain tumor concentration of DGLA did not increased significantly, although a trend toward higher level of DGLA observed in GLAO group. In another study on neonatal rats, addition of 2.9 g GLA/100 g to a formula with low level of DHA and feeding it for about two weeks had no significant effects on DGLA and arachidonic acid levels of rat's forebrain but increased C22: 4 n-6 (adrenic acid) level of brain [34]. We did not measure C22:4 n-6 levels in tumor or serum of rats but tumor and serum levels of DHA in GLAO group were slightly lower than SFO group and it can be speculated that some GLA had been converted to C22:4 n-6 and this fatty acid had replaced some DHA of tumors.

A number of pathways have been suggested as potential contributors to brain PUFA accretion [13]. Blood-brain barrier functions to selectively transport long chained PUFAs into the brain and facilitated transport by FABP7 may be one of the mechanism for this transport [13]. Recombinant FABP7 has high binding affinity for PUFAs than saturated and monounsaturated fatty acids [17,35] and among PUFAs, it exhibits higher affinity for n-3 PUFAs such as DHA and EPA [36]. In fact, binding affinity of DHA to FABP7 is the highest reported among FABPs/ligand interaction [17]. Increase of n-3 fatty acid content of brain tumor tissues in present study accompanied by increase in mRNA levels of FABP7. In addition to their effects on fatty acids transport, FABPs have been shown to influence cellular growth and differentiation [37-39]. FABP7 expression in normal and breast tumor biopsies was associated with mammary gland differentiation and its overexpression in human breast cancer cells induced differentiation [40]. In brain tumor specimens, expression of FABP7 in primary glioblastoma was inversely associated with survival in a specific age group [20] and nuclear but not cytoplasmic FABP7 protein was associated with EGFR overexpression and shorter survival in glioblastoma patients [21]. Study of human glioma cell lines indicate that FABP7 mRNA and protein expression were positively correlated with glial fibrilary acidic protein which is a marker of differentiation of astrocytes [19]. Transfection of FABP7 to FABP7 negative glioma cells decreased proliferation and cell transformation and increased cell differentiation and migration properties of cells, and FABP7 depletion in FABP7 positive glioma cells had no effect on proliferation but decreased migration and resulted in more transformed phenotype [41]. In our study, although DHAO feeding increased FABP7 mRNA levels of rat's brain tumors, it did not affect EGFR expression. Studies on human breast tumor cells have shown that DHA and EPA could actually decrease membrane lipid raft EGFR protein level [42] and mediate part of growth inhibitory effects of DHA on tumor cells [40]. FABPs may also bind PUFAs and decrease their availability to intracellular oxidative pathways [43,44]. Since long-chained PUFAs decrease tumor proliferation by increasing free radical production [45-47], increase in FABP7 expression of glioma following DHA supplementation could reduce prooxidant effect of DHA in tumors and consequently reduce their anti tumor effects. This hypothesis could be explored in future studies.

Since PUFAs are natural ligands of RXRα and PPARγ [22,23], effects of DHAO or GLAO supplementation on gene expression of these two receptors in rat glioma tumors were explored. When compared to SFO, neither DHAO nor GLAO had any significant up or down-regulatory effects on expression of these receptors. One of possible reasons for this observation is large variability of gene expression data and another reason may be short-term intervention of this study. In an study, feeding of fish oil or corn oil to rats increased colon mucosa RXRα and PPARγ mRNA of fish oil group at week 16 but had no significant effects at week 10 [48]. Since in the present study, implantation of C6 cells to rat's brain caused some mortality after 2 weeks, rats were sacrificed at week 2. Because of smallness of developed tumors, we analyzed tumor fatty acids and gene expression in two separate sets of animals in each intervention group and this was another limitation of the current study.

## Conclusion

Supplementation of DHA containing oil could increase long chain n-3 fatty acid content of brain tumors and part of this increase may be mediated by increase in expression of FABP7. Short-term oral consumption of GLA may not be an effective way to increase GLA or DGLA concentrations of brain tumors. Since previous studies have found that enriching tumors with long-chain n-3 fatty acids could have beneficial effects on the treatment of tumors [49], results of present study implicate that oral supplementation is an effective way to increase levels of these fatty acids in brain tumors.

## Methods

### Materials

Docosahexaenoic acid ethyl ester oil (containing 80% DHA) and gamma-linolenic acid ethyl ester oil (containing 72% GLA) were obtained from Minami Nutrition Company (Edegem, Belgium). C6 rat brain glioma cell line was purchased from national cell bank of Iran. Alpha cellulose, Casein, L-Cystin, Cholin bitartrate, tert-butyl hydroquinone, Supelco 37 component fatty acid methyl ester mix, Dulbecco's modified Eagle's Medium (DMEM), Penicillin, streptomycin and Hamilton syringe were obtained from Sigma (Germany, GmbH). Fetal calf serum and trypsin-EDTA were purchased from Invitrogen (Germany, GmbH). RNA Latter, RNeasy mini kit, QuantiTec Reverse transcription kit and Quantifast SYBR green kit were from Qiagen Company (Germany, GmbH). Primers were synthesized by BIONEER (Korea).

### Animals and experimental groups

Female virgin random-bred wistar rats, weighing 200 to 240 g were obtained from Iran Pasteur institute and were studied under the protocol approved by Tehran University of Medical Sciences Animal Research Committee. Rats of each group were housed in different separate cages under temperature of 20 ± 4°C and a 12-h light-dark cycle with free access to food and water.

Since developed tumors from injected C6 cells are small, we decided to determine fatty acid composition in one set of rats and mRNA level in another set of rats in each group. Animals were randomly divided into three groups of twenty seven for SFO, twenty seven for DHAO and thirty for GLAO groups. A set of nine rats for SFO and DHAO and 12 rats for GLAO were randomly selected for analysis of fatty acid composition and remaining animals were assigned for mRNA expression quantitation. A low fat diet (table 3) prepared twice weekly, kept at 4°C and was given to the rats of all groups. Vitamin and mineral mixes used for diet preparation formulated according to AIN93M vitamin and mineral mix [50]. Experimental oils were fed to the animals through a gavage needle at the dose of 1 ml/rat/day. Safflower oil with high linoleic acid (SFO group) and gamma-linolenic acid ethyl ester rich oil (GLAO group) and docosahexaenoic acid rich oil (DHAO) were fed to rats of each groups (table 4). To equalize DHA content of DHAO to the level of GLA in GLAO, 85 volume of DHA 80% was mixed with 15 volume of safflower oil. Diet and oil feeding started one week prior to tumor implantation and continued for 2 weeks after implantation when animals were anesthetized by chloroform inhalation, non-fasting blood was taken from heart and whole brain was extracted, put in RNA Later for RNA extraction or its tumor immediately isolated and snap freezed in liquid nitrogen and kept at -70°C until fatty acid analysis. Brains in RNA later were kept at 4°C for 3–5 days until its tumor area isolated for RNA extraction.

**Table 3:** Composition of low fat diet

**Ingredients**	**g/kg diet**
Corn starch	645

Casein	140

Sucrose	100

Soybean oil	15

Alpha-cellulose	50

Mineral Mix *	35

Vitamin Mix *	10

L-Cystine	1.8

Choline Bitartrate	2.5

Tert-butyl hydroquinone	0.003

*-Formulated according to AIN93M vitamin and mineral mix [50]

**Table 4:** Major fatty acids of oils^1^

**Fatty acids (%)**	**Oil source**		
	
	**High linoleic acid safflower oil (SFO)^2^**	**Gamma-linolenic acid oil (GLAO)**	**Docosahexaenoic acid oil (DHAO)^3^**
Oleic acid	11	0	1.6

Linoleic acid	74–77	26	11.5

Gamma-linolenic acid	0	72	0

Docosahexaenoic acid	0	0	73

1 Refers to fatty acids composition as reported by the supplier.

2 High linoleic President's Choice Safflower Oil

3 Eighty-five volume of DHA 80% Ethyl Ester were mixed with 15 volume of safflower oil to reduce DHA content of oil to 72–74%

### Tumor implantation

C6 rat glioma cell line was grown in DMEM supplemented with 10% (vol/vol) fetal calf serum, 100 mg/ml streptomycin and 100 U/ml penicillin in a 5% CO_2 _atmosphere. At the day of tumor implantation, 85–90% confluent cells were trypsinized, washed by DMEM, counted by hemocytometer and resuspended in DMEM at density of 20 × 10^6^/ml. Animals were anesthetized by an intraperitoneal injection of ketamin 80 mg/kg and xylazine 2 mg/kg prior to surgery and the anesthetized rat was placed in a stereotactic head frame where head was prepped with povidone iodide (10%) and a midsaggital scalp incision was made and after retracting periosteom, a hole was drilled at anteroposterior +3.0 mm, right -2.0 relative to bregma. Tumor cells were injected using a 22 gage Hamilton syringe mounted on the stereotactic holder at the depth of 6 mm. A volume of 5 μL of cell suspension, containing 100 × 10^3 ^cells was injected in 3 minutes and the needle was left in place for 5 minutes. After needle withdrawal, the hole was sealed with sterile bone wax and the incision closed with 4-0 suture.

### Fatty acid extraction and gas chromatography

Fatty acid extraction were carried out by method of Christie [51] with some modifications. Tumor tissue or serum was homogenized in chloroform: methanol (2:1 vol/vol containing 80 mg/L Butylated Hydroxy Toluene) by a glass homogenizer on ice (or vigorous shacking in a glass tube for serum sample), centrifuged and supernatant was taken into a tube. Homogenization repeated twice for the remaining pellet. Normal saline was added to the combined supernatants, shacked vigorously and were allowed to phase separate. The upper layer was drawn off by aspiration followed by addition of methanol: saline (1:1 vol/vol) to the lower phase and the washing procedure was repeated. Extracted lipids were dried under a stream of nitrogen. The dried lipids were soaponified by the method described previously[52]. Soaponified fatty acids were transesterified by boron trifluoride (BF3) in methanol. BF3 was added to the sample and incubated at 100°C in a water bath for an hour. After cooling to room temperature, hexane, HCL and water was added, shacked vigorously, centrifuged and upper phase was taken into a new tube and dried with nitrogen. Before injecting to the instrument, methanol and ethylated margaric acid (as an internal standard) were added to samples and fatty acids methyl esters (FAMEs) measured by gas chromatography.

A capillary column with 60 m length, 0.25 mm internal diameter and 0.2 μM film thickness on a HP 6890 GC equipped with Flame Ionization Detector was used to qualify and quantify FAMEs. The initial column temperature was set at 195°C for 2 min, increased to 205°C by 2°C/min, then to 214°C by 1°C/min, then to 240°C by 15°C/min and held for 10 min. Helium was used as the carrier gas at an initial flow rate of 1 mL/min for 8 min, increased to 1.3 ml/min for 4.2 min and then increased to 1.9 ml/min. The detector temperature was set at 300°C and injector temperature at 250°C. Fatty acids methyl esters were identified by comparison with retention times of Supelco 37 component FAME mix standard. We focused on PUFAs in chromatogram and excluded short and medium chain saturated fatty acids from chromatogram. Different concentrations of FAME mix with added ethylated margaric acid were injected to GC to obtain standard curve for each fatty acid. The peak area of a given fatty acid divided to peak area of internal standard (ethylated margaric acid) was calculated and with regard to standard curve, concentrations of fatty acids in tissue or serum were estimated.

### QRT-PCR

Total RNA was extracted from tumor samples using RNeasy mini kit as per manufacturer's instructions. RNA extract was analyzed spectrophometrically to determine purity and concentration and electrophoresd to check integrity. cDNA was synthesized from extracted RNA using QuantiTect Reverse Transcription Kit in which genomic DNA is removed by genomic DNA wipeout buffer before RT reaction. Synthesized cDNA was analyzed spectrophotometrically to determine concentration and stored in aliquots at -20°C until use.

Table 5 summarized primers used in this study. Primer sequence for PPARγ [53], EGFR [54] and RXRα [55] and FABP7 [56] were obtained from previous studies. Primer sequence for Glyceraldehyde-3-phosphate dehydrogenase (GAPDH) and Beta-Actin were designed by PerlPrimer [57] and OligoAnalyzer (Integrated DNA Technologies), and the specificity of the sequences was analyzed by the BLAST database. Except for GAPDH, the primers were either located in different exons or across exon-exon boundaries.

**Table 5:** Primers for Real-time RT-PCR

**Gene**	**Primer orientation**	**Neucleotide sequence**	**Amplicon **(bp)	**NCBI Accetion number**
Beta-actin	forward	5-CCCTAGACTTCGAGCAAGAG-3	155	NM_031144.2
			
	reverse	5-GGATTCCATACCCAGGAAGG-3		

EGFR	forward	5-CCCACAGCAAGGCTTCTTCA-3	119	NM_031507.1
			
	reverse	5-CACGGCAGCTCCCATTTCTA-3		

FABP7	forward	5-ATGGAGACAAGCTCATTCATGTG-3	134	NM_030832
			
	reverse	5-TGCCTTTTCATAACAGCGAACA-3		

GAPDH	forward	5-GTGCTGAGTATGTCGTGGAGTCTA-3	144	NM_017008.3
			
	reverse	5-TCTCGTGGTTCACACCCATCAC-3		

PPARγ	forward	5-CTGACCCAATGGTTGCTGATTAC-3	80	NM_013124.2
			
	reverse	5-GGACGCAGGCTCTACTTTGATC-3		

RXRα	forward	5-GAGGACATGCCTGTAGAGAAGATT-3	126	NM_012805.2
			
	reverse	5-ACAGATGTTGGTAACAGGGTCATT-3		

Real-time PCR was performed on a Corbett RotorGene 6000 (Corbett Research, Australia) using Quantifast SYBR-green PCR Master Mix. The PCR reactions contained 5 μL of 2X SYBR master mix, 250 ng of cDNA, and 300 nM of GAPDH, Beta-Actin, FABP7, RXR-α primers or 1 μM of EGFR or PPARγ primers in total volume of 10 μL and samples were run in duplicate. Thermal cycler conditions included holds for 5 minutes at 95°C, followed by 43 cycles of 15 seconds at 95°C and 40 seconds at 60°C.A melting curve analysis was performed for each reaction with 62–95°C ramp. No template control (NTC) consisting of H_2_O for target and reference genes were included in each run. Initially, electrophoresis on agarose gel were also performed to demonstrate that qRT-PCR yielded a unique band. The amplification efficiencies of target and reference genes were determined by dilution method, using 3 concentrations of cDNAs of 100, 150 and 250 ng for each gene. Three standard curve of three combined cDNAs of samples were drawn separately and mean efficiency for each gene determined and used for data analysis by *Relative expression software tool (REST) *[58]. Beta-actin and GAPDH served as reference genes.

### Statistical analysis

Concentrations of fatty acids in serum and tissue tested for normality with non-parametric Kolmogorov-Smirnov and variables with no normal distribution were transformed to Lg10 values. One-Way ANOVA was carried out to analyze fatty acids concentrations with *post-hoc *Tukey's multiple comparisons. Two-sided P values < 0.05 were considered significant. The statistical significance of QRT-PCR data calculated using the relative expression software tool which uses pair wise fixed reallocation randomization test. The level of probability was set at *P *< 0.05 as statistically significant.

## Competing interests

The authors declare that they have no competing interests.

## Authors' contributions

JN carried out the design and performed all experiments and prepared the manuscript. FS and MD provide assistance in the design and coordination of the study. MRE helped the statistical analysis. MHM coordinated real time PCR experiment. FSh coordinated cell culture experiment. JMA helped in RNA extraction, cDNA synthesis and real time PCR analysis. ANM dissected brain tumors. KhA carried out GC analysis. SMK coordinated implantation of tumor cell into the brain. All authors have read and approved the content of the manuscript.
